# Case report: Fournier’s gangrene occurring after inavolisib-induced hyperglycemia in a patient with metastatic breast cancer

**DOI:** 10.3389/fphar.2026.1953086

**Published:** 2026-10-28

**Authors:** Pedro Gonzalez Santa-Catalina, Jorge Feito, Sofía Espinal-Matos, María Belén Pazmiño-Zambrano, Romina Pinto-Valdivia, Mar Abad, Emilio Fonseca-Sánchez, César A. Rodríguez-Sánchez, Luis Figuero-Pérez

**Affiliations:** 1 Medical Oncology Department, Salamanca University Hospital, Salamanca, Spain; 2 Biomedical Research Institute of Salamanca (IBSAL), Salamanca, Spain; 3 Faculty of Medicine, University of Salamanca, Salamanca, Spain; 4 Pathology Department, Salamanca University Hospital, Salamanca, Spain

**Keywords:** case report, Fournier’s gangrene, hyperglycemia, inavolisib, PI3K inhibitor

## Abstract

**Background:**

Inavolisib is a selective inhibitor and degrader of mutant phosphatidylinositol 3-kinase alpha (PI3Kα) approved for *PIK3CA*-mutated, hormone receptor-positive, HER2-negative advanced breast cancer, and hyperglycemia is its most frequent class effect. Fournier’s gangrene is an uncommon necrotizing infection of the perineal region with reported mortality of 20%–40%. Necrotizing infections have only exceptionally been described in the context of PI3K inhibitor therapy, but to our knowledge Fournier’s gangrene has not been previously reported with any PI3Kα inhibitor.

**Case Summary:**

A 57-year-old woman with *PIK3CA*-mutated metastatic breast carcinoma, obesity, and impaired fasting glucose without diabetes, receiving fulvestrant, palbociclib and inavolisib, developed grade 3 hyperglycemia refractory to metformin and rapid escalation of basal insulin, with a peak plasma glucose of 623 mg/dL. Inavolisib was interrupted and briefly reintroduced at a reduced dose, whereupon hyperglycemia recurred. Forty-seven days after inavolisib initiation, she was admitted to the intensive care unit with septic shock associated with necrosis of the skin over the perianal region and both buttocks. Contrast-enhanced computed tomography, polymicrobial cultures, and histology confirmed the diagnosis of Fournier’s gangrene. She required surgical debridements and fecal diversion, gradually recovering from the infection and undergoing reconstructive coverage of the gluteal defect. Eight months after presentation, the defect remained durably healed, while the metastatic breast cancer progressed and required two subsequent lines of systemic therapy.

**Conclusion:**

This report describes the occurrence of inavolisib-induced hyperglycemia and a subsequent life-threatening necrotizing infection. Our findings support individualized metabolic and infectious risk stratification before PI3Kα inhibition and urgent evaluation of perineal symptoms in patients who develop severe or refractory hyperglycemia under these targeted therapies.

## 1 Introduction

Activating mutations in *PIK3CA*, the gene that encodes the p110α catalytic subunit of PI3Kα, occur in approximately 40% of hormone receptor (HR)-positive, human epidermal growth factor receptor 2 (HER2)-negative breast carcinomas and are associated with endocrine therapy resistance and adverse prognosis (And et al., 2020; Sobhani et al., 2018; Miller et al., 2011). Inavolisib is an oral agent that selectively inhibits PI3Kα and promotes degradation of the mutant p110α subunit (Hanan et al., 2022). In INAVO120, a phase III, randomized, double-blind, placebo-controlled trial, the combination of inavolisib with palbociclib and fulvestrant extended progression-free and overall survival compared with placebo plus palbociclib and fulvestrant in *PIK3CA*-mutated advanced HR-positive, HER2-negative breast cancer (Turner et al., 2024; Jhaveri et al., 2025).

Hyperglycemia is the most frequent class effect of PI3Kα inhibition (Jhaveri et al., 2026). Insulin signaling proceeds through PI3K and AKT to translocate the GLUT4 transporter, increase peripheral glucose uptake, and suppress hepatic gluconeogenesis; pharmacological blockade of PI3K interrupts this cascade and induces acute insulin resistance with consequent hyperglycemia (Goncalves et al., 2018; Liu et al., 2022). Severity differs across agents directed at this pathway: the incidence of all-grade hyperglycemia ranges from 13.2% with everolimus to 63.7% with alpelisib. This variability largely reflects isoform selectivity, as hyperglycemia is essentially restricted to α-isoform and pan-PI3K inhibitors, given the role of PI3Kα in insulin signaling in adipocytes and myotubes, whereas β-, δ-, and γ-isoform PI3K inhibitors are not implicated (Hee et al., 2026). Nonetheless, the magnitude of the effect in routine clinical practice, along with its capacity to precipitate downstream complications, remains incompletely characterized (Sriravindrarajah et al., 2024).

Fournier’s gangrene is a necrotizing infection of the perineal, genital, and perianal soft tissue, polymicrobial in origin (Hagedorn and Wessells, 2017; Hua et al., 2023). Mortality ranges from 20% to 40% across series (El-Qushayri et al., 2020), and population data indicate higher case fatality among women than men (Abbasi et al., 2025). Diabetes, hyperglycemia, and obesity are recognized as predisposing conditions (Hua et al., 2023). We describe a patient in whom Fournier’s gangrene followed this metabolic toxicity, situate the case against the safety profiles of alpelisib and capivasertib, and interrogate spontaneous reporting data to determine whether the association has any precedent. We present this case in accordance with the CARE reporting checklist (Supplementary Table S1).

## 2 Case description

A 57-year-old postmenopausal, Caucasian woman with a history of metabolic syndrome, obesity (body mass index 38 kg/m^2^, waist circumference 118 cm), active smoking, and deep vein thrombosis, with no known diabetes mellitus, presented after self-detecting a lump in her left breast. She had no family history of diabetes mellitus or of breast or ovarian cancer (germline testing not performed). She was living independently and in active employment at diagnosis, with no barriers to treatment adherence or follow-up. The diagnostic workup that followed led to breast-conserving surgery for a grade 3 infiltrating ductal carcinoma of the left breast, estrogen- and progesterone-receptor positive (70% and 5%, respectively), HER2-ultralow (immunohistochemistry score 0 with faint, incomplete membrane staining in ≤10% of tumor cells), with a Ki-67 proliferation index of 40%, classified as pT2 pN1 M0 (anatomic stage group IIB according to the American Joint Committee on Cancer [AJCC] staging system, 8^th^ edition). To guide the adjuvant chemotherapy decision, the PROSIGNA (PAM50) assay was performed, and it returned a risk-of-recurrence score of 39 and luminal B intrinsic subtype. She subsequently received adjuvant treatment with letrozole, chemotherapy (four cycles of epirubicin 90 mg/m^2^ and cyclophosphamide 600 mg/m^2^ every 2 weeks, followed by eight weekly cycles of paclitaxel 80 mg/m^2^), and radiation therapy (40.05 Gy to the left breast and draining nodal chains with a simultaneous integrated boost of 48 Gy to the tumor bed).

Computed tomography (CT) and positron emission tomography (PET)-CT scans detected metastatic disease recurrence as multiple bone lesions. Real-time PCR for *PIK3CA* mutations, performed on the lumpectomy specimen using the Cobas mutation kit (covering exons 1, 4, 7, 9, and 20 of *PIK3CA*), identified the activating, oncogenic mutation p. (E542K). Then she began fulvestrant plus palbociclib 125 mg, to which inavolisib 9 mg was subsequently added.

Before inavolisib was added, the patient had developed grade 2 neutropenia (Common Terminology Criteria for Adverse Events [CTCAE] version 5.0), which required withholding of palbociclib for 1 week. She had impaired fasting glucose (plasma fasting glucose 110–120 mg/dL, glycated hemoglobin [HbA1c] 5.6%) but no known diabetes mellitus. Oral glucose tolerance test was not performed, and she reported no history of gestational diabetes mellitus). Metformin had been pre-emptively started at 850 mg/12 h, up-titrated to 1,000 mg/12 h when inavolisib 9 mg was initiated. After inavolisib initiation she developed grade 3, persistent hyperglycemia: fasting glucose rose to 348 mg/dL within 9 days, prompting the addition of insulin glargine U300 16 IU/day (0.16 IU/kg/day), and peaked at 623 mg/dL despite rapid up-titration to a ceiling of 50 IU/day (0.52 IU/kg/day). Inavolisib was managed by interruption rather than dose reduction below 6 mg, with a brief reintroduction at 6 mg after glucose normalization, which was followed by recurrence of hyperglycemia, although of lesser severity than at 9 mg (peak 375 mg/dL), and definitive withholding thereafter (the dose was not reduced to 3 mg). Adherence to oral therapy was assessed at each scheduled visit by patient report, and tolerability was graded prospectively using CTCAE version 5.0; adherence was complete throughout the exposure period (Figure 1). Causality between inavolisib and hyperglycemia was graded as “definite” on the Naranjo adverse drug reaction probability scale (Naranjo et al., 1981) (Supplementary Table S2).

**FIGURE 1:**
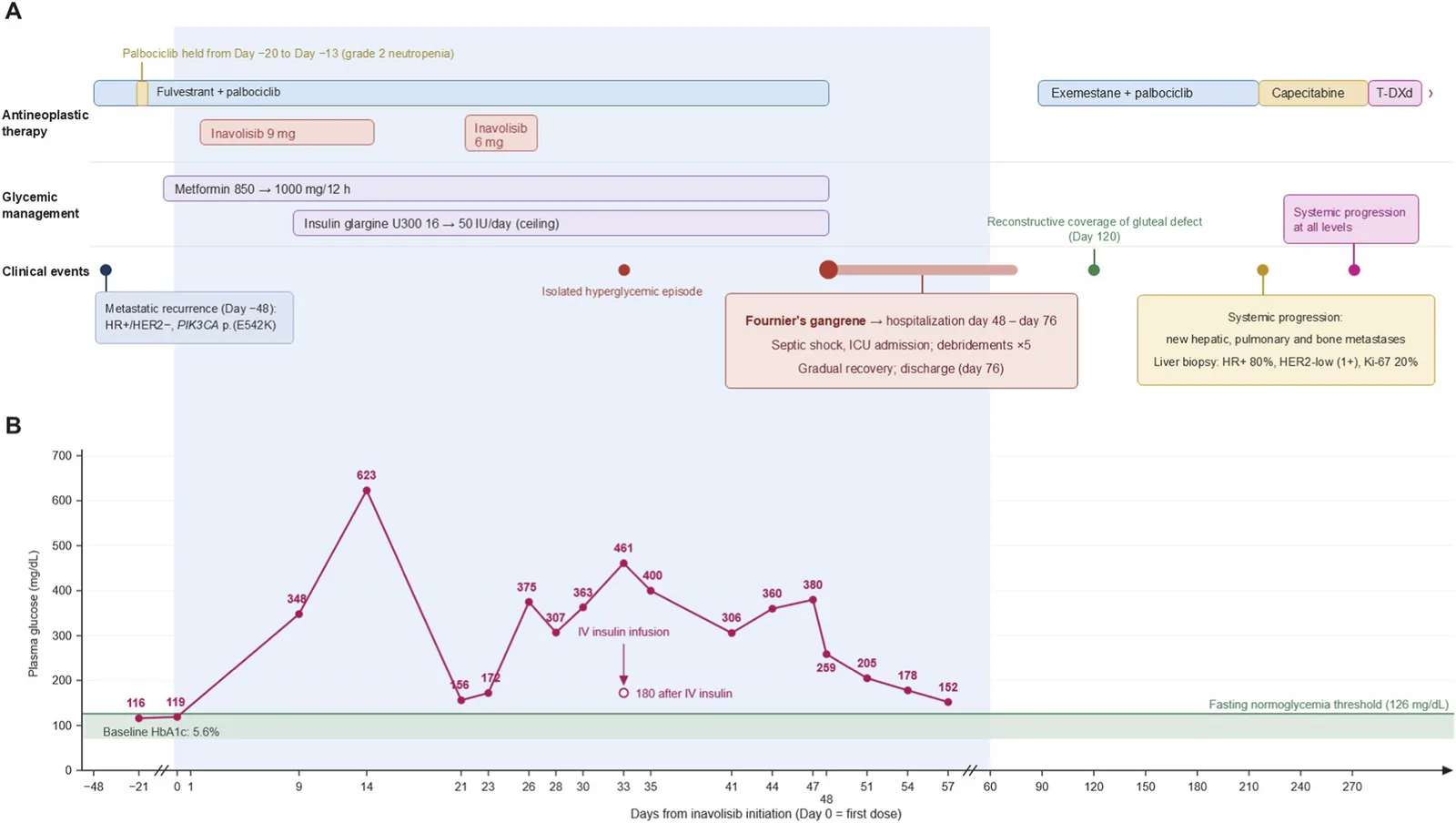
Clinical course of the patient. **(A)** Antineoplastic therapy, glycemic management, and clinical events. **(B)** Recorded plasma glucose concentrations. Both panels share a discontinuous time axis: the early treatment and admission period is displayed at daily resolution, whereas the subsequent follow-up period is compressed to monthly intervals; axis breaks are marked by double oblique strokes, and the expanded interval is indicated by the shaded background. In panel **(A)**, horizontal bars denote the duration of each exposure. Circular markers denote discrete clinical events, and the horizontal bar spanning the admission indicates its duration. In panel **(B)**, markers represent individual recorded determinations and connecting segments join consecutive values; they do not denote continuous monitoring, and intervals without markers were not sampled. Glucose values after normalization during the Fournier’s gangrene admission are not plotted, since no further hyperglycemic crises occurred and monitoring was de-escalated. HER2, human epidermal growth factor receptor 2; HR, hormone receptor; ICU, intensive care unit; IU, international units; *PIK3CA*, phosphatidylinositol-4,5-bisphosphate 3-kinase catalytic subunit alpha (gene); T-DXd, trastuzumab deruxtecan; U300, insulin glargine 300 units per milliliter.

The patient presented to the emergency department with polyuria, polydipsia and asthenia in the context of an isolated hyperglycemic episode (glucose 461 mg/dL, falling to 180 mg/dL after continuous intravenous insulin), and was discharged. She returned 15 days later (48 days after inavolisib was started and 19 days after it was withheld) with malaise and diarrhea. Examination, however, revealed malodorous necrosis of the skin over the perianal region and both buttocks, with erythema, induration, and bullae (Figure 2A); no cutaneous or mucosal portal of entry was identified then or on subsequent imaging. Laboratory studies showed hyperglycemia, acute kidney injury, metabolic acidosis with normal lactate and elevated anion gap, and marked elevation of C-reactive protein, procalcitonin, and creatine kinase, with normal ketonemia and effective serum osmolality (Table 1). She was admitted to the intensive care unit in septic shock and received hemodynamic support and empirical broad-spectrum antibiotics, and contrast-enhanced abdominopelvic CT demonstrated gas within the soft tissue of the perineum and gluteal region (Figures 2B,C). Perianal abscess, non-necrotizing cellulitis of the gluteal skin, and a hyperglycemic emergency were each considered and excluded, the latter by the normal ketonemia and effective serum osmolality. Fournier’s gangrene was diagnosed after multidisciplinary assessment and confirmed by polymicrobial cultures of operative specimens (Supplementary Table S3) and by histology showing vascular thrombosis, coagulative fat necrosis, and a dense neutrophilic infiltrate (Figures 2D–F).

**FIGURE 2:**
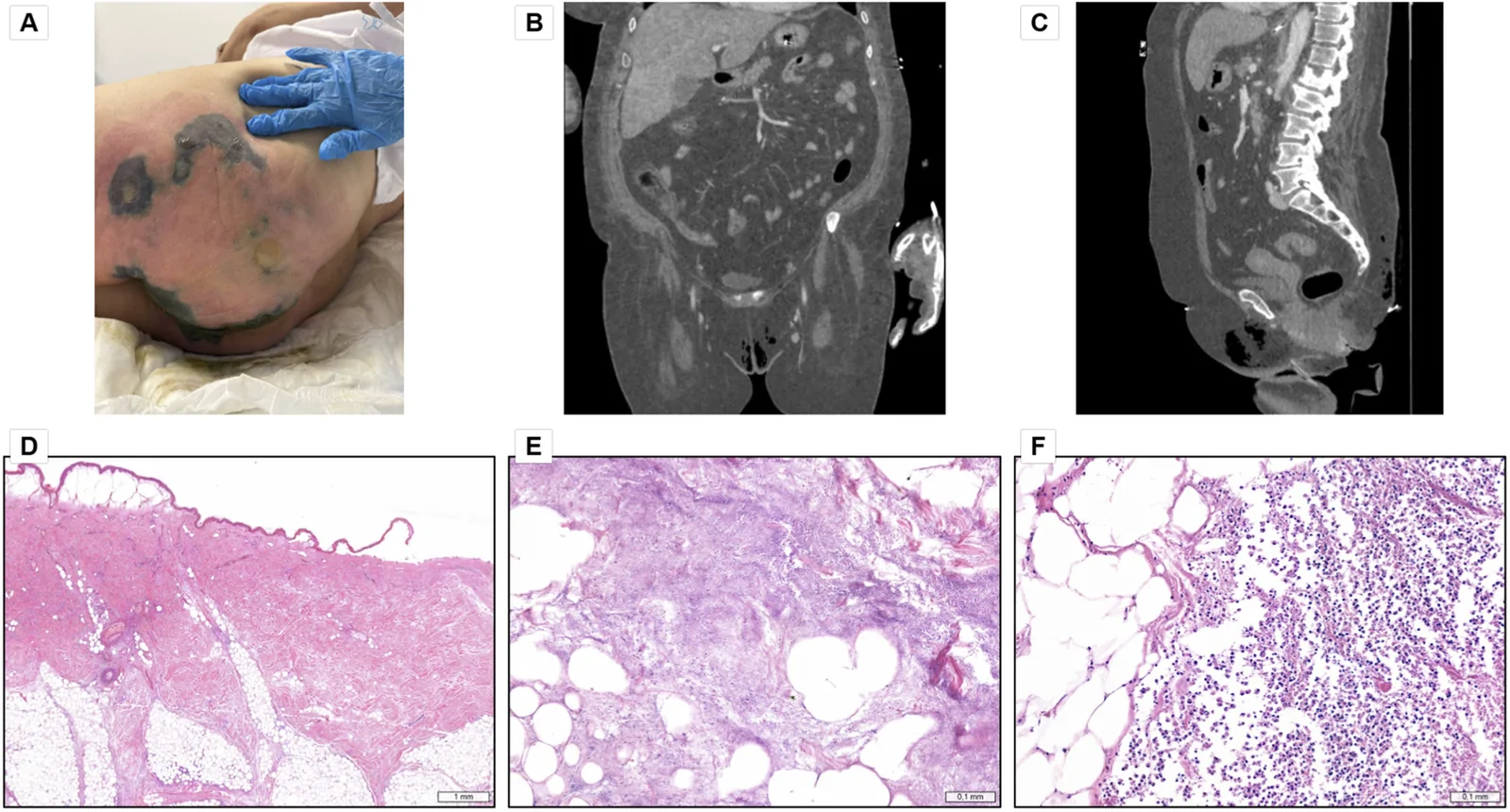
Clinical, radiological, and histological findings of Fournier’s gangrene. **(A)** Macroscopic appearance at presentation: necrosis of the skin over the perianal region and both buttocks, with dark eschar, surrounding erythema and induration, and bulla formation. The image is de-identified. **(B,C)** Contrast-enhanced abdominopelvic computed tomography, coronal **(B)** and sagittal **(C)** reconstructions, demonstrating gas within the subcutaneous and fascial planes of the perineum and gluteal region, a finding consistent with necrotizing infection of soft tissue. **(D–F)** Histological findings in the debridement specimen (hematoxylin and eosin). **(D)** Panoramic view showing full-thickness coagulative necrosis of the dermis extending into the subcutaneous adipose tissue and fascial planes. **(E)** Coagulative necrosis of subcutaneous fat with loss of adipocyte outlines and fibrinoid change of the interlobular septa. **(F)** Dense neutrophilic infiltrate dissecting the necrotic adipose tissue at the advancing edge of the infection.

**TABLE 1:** Laboratory results at presentation to the emergency department with Fournier’s gangrene.

Section	Parameter	Result	Units	Reference range
Arterial blood gas	pH	7.22	​	7.35–7.45
	PCO_2_	39	mmHg	35–45
	Standard bicarbonate	16	mmol/L	22–26
	Base excess	−11	mmol/L	−2 to +2
	Lactate	1.2	mmol/L	0.5–2.0
Biochemistry	Glucose	259	mg/dL	74–106
	Urea	226	mg/dL	15–45
	Creatinine	4.04	mg/dL	0.7–1.3
	eGFR (Cockcroft-Gault)	<15	mL/min	>90
	Phosphate	7.3	mg/dL	2.5–4.5
	Magnesium	1.88	mg/dL	1.7–2.2
	Sodium	138	mmol/L	135–145
	Potassium	3.6	mmol/L	3.5–5.1
	Chloride	103	mmol/L	98–107
	Anion gap	19	mmol/L	8–16
	ALT	76	U/L	7–40
	LDH	264	U/L	120–250
	Total bilirubin	0.8	mg/dL	0.3–1.2
	GGT	54	U/L	8–40
	Alkaline phosphatase	67	U/L	40–130
	Albumin	35	g/L	35–50
	CRP	46.59	mg/dL	0–0.5
	Procalcitonin	26.49	ng/mL	0–0.5
	CK	637	U/L	34–145
	Beta-hydroxybutyrate	<0.6	mmol/L	<0.6
	Effective serum osmolality	290	mOsm/kg	275–295
Blood count	Hemoglobin	9.2	g/dL	12–16
	WBC	7.98	x10^3^/µL	4–11
	ANC	6.98	x10^3^/µL	2–7.5
	ALC	0.57	x10^3^/µL	1–4
	APC	257	x10^3^/µL	150–400
Coagulation	Prothrombin activity	81.5	%	70–120
	aPTT	29.3	s	25–35
	INR	1.09	​	0.8–1.2
	Fibrinogen	>899	mg/dL	200–400
	D-dimer	1.3	mg/L	0–0.5

ALC, absolute lymphocyte count; ALT, alanine aminotransferase; ANC, absolute neutrophil count; APC, absolute platelet count; aPTT, activated partial thromboplastin time; CK, creatine kinase; CRP, C-reactive protein; eGFR, estimated glomerular filtration rate; GGT, gamma-glutamyl transferase; INR, international normalized ratio; LDH, lactate dehydrogenase; PCO_2_, partial pressure of carbon dioxide; WBC, white blood cell count.

Urgent debridement was performed, followed by four further debridements and a laparoscopic colostomy with mucous fistula for fecal diversion; she recovered gradually and was discharged, with reconstructive coverage of the gluteal defect performed subsequently. From the patient’s standpoint, the perineal and gluteal pain resolved, and she returned to independent living and to work (Karnofsky Performance Status: 90 points). Inavolisib was permanently withdrawn because of this toxicity (grade 4 necrotizing fasciitis, according to the CTCAE v5.0 codification).

After ruling out progressive disease on subsequent CT reevaluation, first-line therapy was resumed rather than escalating to chemotherapy: exemestane replaced fulvestrant, chosen given prior disease progression on letrozole and because intramuscular gluteal administration of fulvestrant was no longer suitable after debridement and reconstruction of the gluteal region, and palbociclib was reintroduced in combination, continuing CDK4/6 inhibition (Figure 1). Afterwards, the patient experienced progression of bone lesions and the appearance of multiple hepatic and pulmonary metastases. This prompted a switch to second-line treatment for metastatic disease with capecitabine monotherapy (1,000 mg/m^2^ orally twice daily for 14 consecutive days in 21-day cycles). At the first reassessment on this line of treatment, by PET-CT, further progression of the bone, pulmonary, and hepatic lesions was documented. Core-needle biopsy of one of the hepatic lesions showed HR positivity (80%), a Ki-67 index of 20%, and HER2-low expression (immunohistochemistry score 1+). She is currently receiving third-line treatment with trastuzumab deruxtecan 5.4 mg/kg intravenously every 21 days (Figure 1). At the most recent assessment, 8 months after presentation, the gluteal defect remained durably healed, with no recurrence of soft-tissue infection.

Written informed consent was obtained from the patient for publication of this case report, its accompanying images, and the personal account reproduced in the Patient perspective section.

## 3 Discussion

Hyperglycemia is one of the most frequent on-target class effects of PI3Kα inhibition, reported in a majority of patients receiving inavolisib in INAVO120 (Turner et al., 2024) (Table 2), and well characterized in dedicated series (Jhaveri et al., 2026; Liu et al., 2022). Fournier’s gangrene, in turn, is an uncommon necrotizing infection of the perineum whose mortality remains high across series (El-Qushayri et al., 2020), with a higher case fatality in women (Abbasi et al., 2025). The convergence of these two entities during PI3Kα-directed therapy has not been described in the peer-reviewed literature, and interrogation of spontaneous reporting data, presented below, identified no independent precedent.

**TABLE 2:** Hyperglycemia and necrotizing infection of soft tissue across three PI3K-pathway agents: registrational trial context, trial-reported rates, and FDA adverse event reporting system (FAERS) reports.

​	Inavolisib	Alpelisib	Capivasertib
Registrational trial	INAVO120 (NCT04191499) (Turner et al., 2024; Im et al., 2026)	SOLAR-1 (NCT02437318) (André et al., 2019; Rugo et al., 2020)	CAPItello-291 (NCT04305496) (Turner et al., 2023; Rugo et al., 2024)
Design	Phase III, randomized, double-blind, placebo-controlled	Phase III, randomized, double-blind, placebo-controlled	Phase III, randomized, double-blind, placebo-controlled
Regimen	Inavolisib 9 mg once daily or placebo, each with palbociclib 125 mg on days 1–21 of 28-day cycles and fulvestrant 500 mg	Alpelisib 300 mg once daily or placebo, each with fulvestrant 500 mg	Capivasertib 400 mg twice daily, 4 days on and 3 days off, or placebo, each with fulvestrant, in 4-week cycles
Population	*PIK3CA*-mutated, HR-positive, HER2-negative advanced disease; no prior therapy for advanced disease; recurrence during or within 12 months of adjuvant endocrine therapy	HR-positive, HER2-negative advanced disease progressing during or after aromatase inhibitor therapy; *PIK3CA*-mutated cohort (n = 341) and non-mutated cohort (n = 231)	HR-positive, HER2-negative advanced disease relapsing or progressing during or after aromatase inhibitor therapy, with or without prior CDK4/6 inhibitor; 289 of 708 with *PIK3CA*, *AKT1*, or *PTEN* alterations
Safety population^a^	n = 161 (placebo n = 164)	n = 284 (placebo n = 287)	n = 355 (placebo n = 350)
Glycemic entry criteria^b^	Fasting glucose below 126 mg/dL and glycated hemoglobin below 6.0%	Fasting plasma glucose at or below 140 mg/dL and glycated hemoglobin at or below 6.4%; type 1 diabetes and uncontrolled type 2 diabetes excluded	Patients with insulin-dependent diabetes excluded
Hyperglycemia, any grade	63.4% (placebo 13.5%)	63.7%	16.9%^c^
Hyperglycemia, grade 3 or 4	5.6% (placebo 0%)	36.6% (grade 3, 32.7%; grade 4, 3.9%)	2.3%^c^
Other frequent grade 3 or higher events	Neutropenia 80.2% (placebo 78.4%), thrombocytopenia 14.2%, stomatitis 5.6%, diarrhea 3.7%	Rash 9.9%, diarrhea 6.7%	Rash 12.1%, diarrhea 9.3%
Discontinuation for any adverse event	6.8% for any trial agent (placebo 0.6%)	25.0% (alpelisib)	13.0% (capivasertib)
Discontinuation attributed to hyperglycemia	Not reported separately	6.3%	0.3%
FAERS reports naming the agent as suspect product	470	8,697	2,753
Reports documenting hyperglycemia^d^	29.1%	18.7%	11.7%
Reports of Fournier’s gangrene	4^e^	0	0
Independent cases of Fournier’s gangrene	0	0	0
Other necrotizing infection of soft tissue	0	1 fatal necrotizing fasciitis (January 2023)	0
Cellulitis	0^f^	32	5

AKT1, AKT, serine/threonine kinase 1; CDK4/6, cyclin-dependent kinase 4/6; FAERS, FDA, adverse event reporting system; HER2, human epidermal growth factor receptor 2; HR, hormone receptor; *PIK3CA*, phosphatidylinositol-4,5-bisphosphate 3-kinase catalytic subunit alpha gene; *PTEN*, phosphatase and tensin homolog gene.

^a^ Safety population: all patients who received at least one dose of study treatment, analyzed for adverse events regardless of efficacy population assignment.

^b^ Entry thresholds were not equivalent: INAVO120 applied the most restrictive glycemic ceiling of the three, which limits cross-trial comparison of hyperglycemia frequency.

^c^ Values correspond to the grouped Medical Dictionary for Regulatory Activities (MedDRA) term.

^d^ Public dashboard, cumulative to 27 July 2026. Percentages denote the proportion of spontaneous reports, not incidence: the database lacks a denominator of exposed patients and cannot establish causality.

^e^ All of 4 reports (notified separately by four marketing authorization holders) are attributable to the same single patient.

^f^ Absence confirmed in the openFDA, reaction profile for the generic and brand names, in which no cellulitis report appears among the reported preferred terms.

Hyperglycemia is the common metabolic adverse effect associated with PI3K-pathway inhibitors, but its severity diverges considerably between agents (Turner et al., 2024; Im et al., 2026; André et al., 2019; Rugo et al., 2020; Turner et al., 2023; Rugo et al., 2024) (Table 2). Inavolisib’s mutant-selective inhibition and degradation of p110α may partially spare wild-type metabolic signaling (Hanan et al., 2022), consistent with its low rate of grade 3 or 4 hyperglycemia despite its high any-grade rate. However, this interpretation remains speculative, since cross-trial comparison is confounded by differing populations, glycemic entry criteria, and monitoring intensity.

In our patient, severe, refractory hyperglycemia (plasma glucose 623 mg/dL despite basal insulin at 0.52 IU/kg/day) developed despite inavolisib’s comparatively low population-level rate of grade 3 or 4 hyperglycemia. This discordance likely reflects patient-specific factors rather than an unexplained statistical outlier: pre-existing obesity, impaired fasting glucose, and metabolic syndrome, all present before inavolisib was started, plausibly lowered her threshold for progression to severe hyperglycemia once PI3Kα inhibition was superimposed, converting a typically mild on-target effect into a severe one. Separately, grade 3 or 4 neutropenia in INAVO120 was nearly identical between arms (Table 2) (Turner et al., 2024), which attributes that risk to palbociclib rather than to PI3Kα inhibition. The triplet regimen thus couples a high-frequency hematologic insult with a metabolic insult of variable severity, the two capable of converging on host defense through independent routes, although in this patient neutropenia reached only grade 2 and had resolved before presentation.

The clinical sequence in our patient can be reconstructed mechanistically. First, selective inhibition of the p110α catalytic isoform, a dominant mediator of insulin action in liver, muscle, and adipose tissue, attenuated PI3K/AKT signaling in host metabolic tissues, suppressing GLUT4-dependent glucose uptake and driving insulin resistance and hyperglycemia (Goncalves et al., 2018; Hopkins et al., 2018); compensatory hyperinsulinemia, which in preclinical models can partially reactivate PI3K signaling (Hopkins et al., 2018), plausibly contributed to the refractory, escalating glycemic course, although this cannot be proven from a single case. Second, sustained hyperglycemia impaired neutrophil chemotaxis, phagocytosis, and reactive-oxygen-dependent killing, dampened complement function, and produced microvascular dysfunction, conditions that favor bacterial proliferation in soft tissue (Dowey et al., 2021). Third, these effects acted on a susceptible background of active malignancy, metabolic syndrome, active smoking, and obesity, which independently impairs local tissue perfusion and lymphatic drainage and promotes moisture retention and bacterial proliferation within skinfolds (Ren et al., 2026; Lim et al., 2026), with an earlier episode of palbociclib-induced grade 2 neutropenia as a possible additional contributor; the interval from inavolisib initiation to presentation was only 48 days. Finally, this immunometabolically compromised status culminated in polymicrobial Fournier’s gangrene, for which inadequate glycemic control following PI3Kα inhibition appears to be the most plausible proximate contributor (the sequence is inferred rather than demonstrated).

To corroborate the novelty of our report independently, we interrogated the FDA Adverse Event Reporting System (public dashboard, cumulative to 27 July 2026) for reports naming inavolisib, alpelisib, or capivasertib as suspect product, screening Medical Dictionary for Regulatory Activities (MedDRA) preferred terms for necrotizing infection of soft tissue (FDA Adverse Event Reporting System, 2026). The FAERS report counts, by agent, along with hyperglycemia documentation rates, Fournier’s gangrene reports, and related soft-tissue infection counts, are summarized in Table 2; the hyperglycemia gradient parallels the frequencies reported in the registrational trials, although spontaneous reporting rates are shaped by exposure volume, indication, and reporting behavior and cannot be interpreted as a measure of pharmacological potency. Fournier’s gangrene appeared only in reports naming inavolisib: four entries sharing identical patient data (age, event date, drug combination, and concomitant medication), presumably the same case notified separately by four marketing authorization holders. No independent case of Fournier’s gangrene is registered for any other agent of this class. The absence of any independent case supports the novelty of our description, although tempered by two facts: spontaneous reporting lacks a denominator (the total number of patients exposed, needed to calculate an incidence rate) and cannot establish causality; and inavolisib’s narrow exposure base since its regulatory approval further limits interpretation.

Since severe hyperglycemia preceded this complication in our patient, baseline metabolic status (impaired fasting glucose, elevated glycated hemoglobin, obesity, and insulin resistance) offers the strongest available means of identifying patients at greatest risk of pronounced glycemic excursions during PI3K or AKT inhibition (Liu et al., 2022). Our patient carried three of these four risk factors yet had a normal pretreatment glycated hemoglobin (5.6%), illustrating that a reassuring baseline does not exclude catastrophic decompensation. Because hyperglycemia stimulates insulin release, and insulin can reactivate PI3K signaling in tumor tissue (Hopkins et al., 2018), agents that lower glucose without raising insulin are preferable, with metformin recommended as initial therapy (Liu et al., 2022). Insulin, by contrast, is a rescue measure whose escalation may undercut the rationale for avoiding insulin-driven signaling: in our patient, glycemic control was not achieved even at a 50 IU/day ceiling.

No oral glucose tolerance test was performed before treatment, and there was no history of gestational diabetes mellitus. In patients with obesity and impaired fasting glucose, HbA1c may not capture a pre-existing impairment of glucose tolerance, which reduces the reserve available to absorb the insulin resistance imposed by PI3Kα inhibition (Hee et al., 2026; Lim et al., 2026). An oral glucose tolerance test at baseline would have quantified this reserve and, had it revealed impaired glucose tolerance, might have justified closer surveillance from the first week, a lower threshold for starting basal insulin, or reconsideration of the initial inavolisib dose, even though metformin had already been started pre-emptively. Timing of measurement also matters. Hyperglycemia induced by PI3K inhibitors follows each dose, with glucose peaking within hours of administration and returning toward baseline before the next dose (Hee et al., 2026; Lim et al., 2026), so fasting determinations alone may miss the largest excursions. Monitoring in our patient relied on fasting glucose, and the magnitude of post-dose hyperglycemia during the first weeks may have been underestimated.

Weekly glucose monitoring during the first month, tapering to every 2 weeks through month three, would help detect early decompensation, and perineal or perianal pain accompanied by uncontrolled glucose warrants immediate assessment rather than empirical outpatient antibiotics. Once hyperglycemia is established, published guidance recommends interrupting the drug when fasting glucose exceeds 250 mg/dL despite optimized antidiabetic therapy, with reintroduction at a reduced dose only after values fall below 160 mg/dL (Jhaveri et al., 2026). Our patient followed this approach, yet drug withdrawal reversed the metabolic abnormality without preventing the infectious complication, which appeared 19 days later. Reversibility of a laboratory value therefore offers no guarantee of clinical safety, and the period of greatest vulnerability may precede the decision to interrupt treatment.

This report has two principal strengths: the diagnosis rests on multidisciplinary evaluation with concordant microbiological and histological confirmation, and the temporal course was documented in detail, from baseline impaired fasting glucose to refractory hyperglycemia and necrotizing infection (Figure 1). Its limitations derive from the nature of a single observation. Causality is inferred rather than proven, and the concurrent exposure to antidiabetic agents, alongside a background of predisposing conditions, precludes attribution to any single factor. No local portal of entry was identified, so the initiating event for the perineal infection remains undetermined. Although the Naranjo scale (Naranjo et al., 1981) graded the inavolisib–hyperglycemia relationship as definite, it addresses only the direct drug–reaction link and not the gangrene. Fournier’s gangrene developed 19 days after inavolisib had been permanently discontinued, and at presentation the patient also had acute kidney injury, metabolic acidosis and septic shock, among other aforementioned risk factors for severe infection; therefore, a causal role for inavolisib-induced hyperglycemia in the subsequent infection cannot be established, so hyperglycemia is best regarded as a plausible precipitating factor. These limitations could be overcome by systematic pharmacovigilance reporting and multicenter registry or cohort studies coupled with protocolized prospective glycemic monitoring in patients receiving PI3Kα inhibitors (Jhaveri et al., 2026; Sriravindrarajah et al., 2024), which together would establish whether this association is reproducible.

In conclusion, this case report illustrates how a common, generally manageable class effect can, in routine practice, escalate into a rare and potentially lethal complication, and it underscores the value of individualized metabolic and infectious risk stratification before and during treatment. Clinicians who prescribe inavolisib should anticipate hyperglycemia, monitor glycemic status closely from the outset, treat it aggressively when refractory, and act without delay at the earliest signs of perineal infection.

## 4 Patient perspective

“When my treatment began, I understood that side effects were possible, but the rise in my blood sugar and the infection that followed were far more serious than I had expected. The illness was frightening and the recovery was long, yet I felt supported by the team who cared for me at every stage. I hope that sharing my experience will help doctors recognize this complication early and protect other patients who receive the same treatment”.

## Data Availability

Publicly available datasets were analyzed in this study. This data can be found here: https://fis.fda.gov/sense/app/95239e26-e0be-42d9-a960-9a5f7f1c25ee/sheet/7a47a261-d58b-4203-a8aa-6d3021737452/state/analysis. The clinical data supporting the case description are not publicly available because they contain information that could compromise the privacy of the patient; anonymized data are available from the corresponding author on reasonable request.
