# Supplementary material for: Case report: Fournier’s gangrene occurring after inavolisib-induced hyperglycemia in a patient with metastatic breast cancer
Source: Front Pharmacol. 2026 Oct 28;17:1953086. doi: 10.3389/fphar.2026.1953086 (PMC13752534; doi:10.3389/fphar.2026.1953086)
Supplement: Supplementary file 1 [file Table1.docx]

# CARE Checklist of information to include when writing a case report

**Topic	Item	Checklist item description	Reported on Section**

**Title	1**	The diagnosis or intervention of primary focus followed by the words “case report”	Title

**Key Words	2**	2 to 5 key words that identify diagnoses or interventions in this case report, including "case report"	Keywords

**Abstract**

**(no references)**

**3a**	Introduction: What is unique about this case and what does it add to the scientific literature?	Abstract (Background)

**3b**	Main symptoms and/or important clinical findings	Abstract (Case summary)

**3c**	The main diagnoses, therapeutic interventions, and outcomes	Abstract (Case summary)

**3d**	Conclusion—What is the main “take-away” lesson(s) from this case?	Abstract (Conclusion)

**Introduction	4**	One or two paragraphs summarizing why this case is unique (may include references)	Introduction

**Patient Information	5a**	De-identified patient specific information	Case description (para. 1)

**5b**	Primary concerns and symptoms of the patient	Case description (paras. 1, 4)

**5c**	Medical, family, and psycho-social history including relevant genetic information	Case description (paras. 1-3)

**5d**	Relevant past interventions with outcomes	Case description (paras. 1-3)

**Clinical Findings**

**Timeline**

**Diagnostic Assessment**

**Therapeutic Intervention**

**Follow-up and Outcomes**

6 Describe significant physical examination (PE) and important clinical findings	Case description (para. 4)
7 Historical and current information from this episode of care organized as a timeline	Figure 1

**8a**	Diagnostic testing (such as PE, laboratory testing, imaging, surveys)	Table 1; Figure 2

**8b**	Diagnostic challenges (such as access to testing, financial, or cultural)	Not applicable

**8c**	Diagnosis (including other diagnoses considered)	Case description (para. 4)

**8d**	Prognosis (such as staging in oncology) where applicable	Case description (paras. 1-2)

**9a**	Types of therapeutic intervention (such as pharmacologic, surgical, preventive, self-care)	Case description (paras. 1-6)

**9b**	Administration of therapeutic intervention (such as dosage, strength, duration)	Case description (paras. 1-3, 6)

**9c**	Changes in therapeutic intervention (with rationale)	Case description (paras. 3, 5, 6)

**10a**	Clinician and patient-assessed outcomes (if available)	Case description (paras. 5-6)

**10b**	Important follow-up diagnostic and other test results	Case description (para. 6)

**10c**	Intervention adherence and tolerability (How was this assessed?)	Case description (para. 3)

**10d**	Adverse and unanticipated events	Case description (paras. 3-5)

**Discussion	11a**	A scientific discussion of the strengths AND limitations associated with this case report	Discussion

**11b**	Discussion of the relevant medical literature **with references**	Discussion, Table 2

**11c**	The scientific rationale for any conclusions (including assessment of possible causes)	Discussion

**11d**	The primary “take-away” lessons of this case report (without references) in a one paragraph conclusion	Discussion (final para.)

**Patient Perspective	12**	The patient should share their perspective in one to two paragraphs on the treatment(s) they received	Patient perspective

**Informed Consent	13**	Did the patient give informed consent? Please provide if requested	**Yes** **No**
