# Supplementary material for: Case report: Fournier’s gangrene occurring after inavolisib-induced hyperglycemia in a patient with metastatic breast cancer
Source: Front Pharmacol. 2026 Oct 28;17:1953086. doi: 10.3389/fphar.2026.1953086 (PMC13752534; doi:10.3389/fphar.2026.1953086)
Supplement: Supplementary file 2 [file Table2.docx]

**Supplementary Table 2.** Naranjo adverse drug reaction probability scale for the association between inavolisib and hyperglycemia.

| **Question** | **Yes** | **No** | **Do not know** | **Answer** | **Score** |
| --- | --- | --- | --- | --- | --- |
| 1. Are there previous conclusive reports on this reaction? | +1 | 0 | 0 | Yes | +1 |
| 2. Did the adverse event appear after the suspected drug was administered? | +2 | -1 | 0 | Yes | +2 |
| 3. Did the reaction improve when the drug was withdrawn or an antagonist given? | +1 | 0 | 0 | Yes | +1 |
| 4. Did the reaction reappear when the drug was re-administered? | +2 | -1 | 0 | Yes | +2 |
| 5. Are there alternative causes that could on their own have produced the reaction? | -1 | +2 | 0 | No | +2 |
| 6. Did the reaction reappear with placebo? | -1 | +1 | 0 | Not assessed | 0 |
| 7. Was the drug detected in toxic concentrations? | +1 | 0 | 0 | Not assessed | 0 |
| 8. Was the reaction more severe with a higher dose or less severe with a lower dose? | +1 | 0 | 0 | Yes (less severe with a lower dose) | +1 |
| 9. Did the patient have a similar reaction to the same or a related drug previously? | +1 | 0 | 0 | No | 0 |
| 10. Was the adverse event confirmed by objective evidence? | +1 | 0 | 0 | Yes | +1 |
| **Total score** | | | | | **+10** |

Adverse drug reaction probability scale is: ≥9, definite; 5–8, probable; 1–4, possible; , ≤0, doubtful (16).
