# Supplementary material for: Case report: Fournier’s gangrene occurring after inavolisib-induced hyperglycemia in a patient with metastatic breast cancer
Source: Front Pharmacol. 2026 Oct 28;17:1953086. doi: 10.3389/fphar.2026.1953086 (PMC13752534; doi:10.3389/fphar.2026.1953086)
Supplement: Supplementary file 3 [file Table3.docx]

**Supplementary Table 3.** Microbiological culture and susceptibility from operative debridement.

| **Bacteria** | 1. *Klebsiella pneumoniae*  2. *Escherichia coli*  3. *Bacteroides ovatus*  4. *Streptococcus constellatus* | | | |
| --- | --- | --- | --- | --- |
| **Antibiotics** | **1** | **2** | **3** | **4** |
| Amoxicillin-clavulanate | ‒ | ‒ | R | ‒ |
| Piperacillin-tazobactam | S | S | ‒ | ‒ |
| Trimethoprim-sulfamethoxazole | S | S | ‒ | ‒ |
| Ceftriaxone | S | S | ‒ | ‒ |
| Metronidazole | ‒ | ‒ | S | ‒ |
| Penicillin | ‒ | ‒ | ‒ | S |

S, susceptible; R, resistant; ‒, not performed.
